# Database of daily Lagrangian Arctic sea ice parcel drift tracks with coincident ice and atmospheric conditions

**DOI:** 10.1038/s41597-023-01987-6

**Published:** 2023-02-04

**Authors:** Sean Horvath, Linette Boisvert, Chelsea Parker, Melinda Webster, Patrick Taylor, Robyn Boeke, Steven Fons, J. Scott Stewart

**Affiliations:** 1 NASA Goddard Space Flight Center, 8800 Greenbelt Rd., Greenbelt, MD 20771 USA; 2 Earth System Science Interdisciplinary Center, University of Maryland, 5825 University Research Court Suite 4001, College Park, MD 20740 USA; 3 University of Alaska Fairbanks, Geophysical Institute, 2156 Koyukuk Drive, Fairbanks, AK 99775 USA; 4 NASA Langley Research Center, Climate Science Branch, Hampton, VA 23681 USA; 5 Science Systems Applications Inc., Hampton, VA 23666 USA; 6 National Snow and Ice Data Center, Boulder, CO 80309 USA; 7 Polar Science Center, University of Washington, Seattle, WA 98105 USA

**Keywords:** Cryospheric science, Physics

## Abstract

Since the early 2000s, sea ice has experienced an increased rate of decline in thickness, extent and age. This new regime, coined the ‘New Arctic’, is accompanied by a reshuffling of energy flows at the surface. Understanding of the magnitude and nature of this reshuffling and the feedbacks therein remains limited. A novel database is presented that combines satellite observations, model output, and reanalysis data with sea ice parcel drift tracks in a Lagrangian framework. This dataset consists of daily time series of sea ice parcel locations, sea ice and snow conditions, and atmospheric states, including remotely sensed surface energy budget terms. Additionally, flags indicate when sea ice parcels travel within cyclones, recording cyclone intensity and distance from the cyclone center. The quality of the ice parcel database was evaluated by comparison with sea ice mass balance buoys and correlations are high, which highlights the reliability of this database in capturing the seasonal changes and evolution of sea ice. This database has multiple applications for the scientific community; it can be used to study the processes that influence individual sea ice parcel time series, or to explore generalized summary statistics and trends across the Arctic.

## Background & Summary

Drastic changes occurring in the Arctic sea ice cover in recent years^1–4^ have been a topic of great concern not only for the scientific community and local inhabitants, but also for the general public, policymakers and stakeholders. This is because ‘what happens in the Arctic does not remain in the Arctic’ but rather is connected to areas at lower latitudes^5,6^. Changes in the Arctic will have profound effects politically, economically, ecologically, and climatologically on Earth. The Arctic is experiencing the largest temperature increases on our planet^7^ due to global warming. This process is attributed to Arctic Amplification^8–10^ and is driving the rapid changes in the Arctic. The most striking change is the decline in Arctic sea ice extent since the late 1970s^11,12^. Since the early 2000s, sea ice has experienced an increased rate of decline in thickness and volume, and transitioned to a predominantly seasonal ice cover^2,13,14^ compared to a perennial ice cover in the 1980–1990s^15–17^. From 2000 onward, observations suggest that the Arctic has become warmer and wetter^18^, evaporation and turbulent fluxes from the ice-free ocean has increased^19,20^, the surface albedo has darkened^21^, and cloud cover has also increased^22,23^. This era with these large changes observed in the Arctic climate system has been coined the ‘New Arctic’.

The shift to thinner, seasonal ice in the ‘New Arctic’ is accompanied by a reshuffling of energy flows at the surface^6^. Understanding of the magnitude and nature of the reshuffling of the Arctic surface energy budget (SEB) and the feedbacks therein remains limited. This knowledge gap is illustrated by the large spread in climate model projections of the changes in surface turbulent fluxes, near surface temperatures, and hence lower tropospheric stability^20,24,25^. The temperature structure of the lower atmosphere and changes in the SEB (induced by the changes in the sea ice pack) are leading drivers of Arctic Amplification^10,24–26^. Therefore, synthesizing observations to better understand the evolution of the lower tropospheric temperature structure and its influence on the SEB is critical for improving model predictive capabilities of Arctic Amplification and future sea ice change.

Sea ice growth and melt are driven by changes in the SEB, which is influenced by atmospheric forcing and climate variability^25,27,28^. Studies have recently shown that the accumulation of radiative energy at the surface in early summer (June, July, and August) is a good predictor of September sea ice extent (i.e., sea ice survival)^29^. Sea ice thickness has been shown to be important for predicting September sea ice area up to 6 months in advance^30^ owing to both thickness and extent being influenced by the same thermodynamic and dynamic processes. Thus, understanding what drives the year-to-year variability of sea ice thickness and extent, through winter preconditioning and melt season evolution, can help elucidate the drivers behind different projected trends in Arctic sea ice loss.

A quantitative understanding of the interaction between sea ice and the atmosphere is important for describing the coupled Arctic climate system and is necessary for improving model physics, which, in turn, can improve seasonal forecasts and climate projections of the fate of the sea ice in the ‘New Arctic’. Previous studies have examined and quantified sea ice-atmospheric interactions using an Eulerian framework^31–33^. However, given sea ice mobility, this is a serious limitation to process-oriented understanding by inhibiting the ability to track cumulative effects of atmospheric processes on the SEB and sea-ice mass balance. Studying sea ice from a Lagrangian framework has been used for tracing biogeochemical transport^34,35^, ice volume flux^36–38^, snow distribution^39^, and for developing a numerical sea ice model^40^. Lagrangian tracking of coincident sea ice and atmospheric conditions has also been done^41^, which we expand on here with higher temporal resolution (daily) and more complete atmospheric conditions including terms for calculating the SEB. In this work, we present the creation of a database to monitor the memory of the sea ice parcels using a Lagrangian framework, tracking their daily motion, characteristics such as thickness and concentration, SEB, and associated atmospheric conditions as they undergo seasonal evolution and drift through the Arctic Ocean between October 2002 and September 2020. The database starts in 2002 as this highlights conditions in the ‘New Arctic’ and due to availability of important satellite data. This framework will enable the scientific community to effectively monitor and analyze the evolution of the sea ice and SEB over a variety of atmospheric and sea ice conditions. This effort uniquely unifies a wide variety of satellite and reanalysis data and can provide crucial knowledge of how the ‘New Arctic’ sea ice couples with the atmosphere, and also how a range of atmospheric forcings and episodic weather events influence the SEB, sea-ice mass balance, and hence seasonal evolution of Arctic sea ice.

## Methods

### Lagrangian tracked sea ice parcels

Sea ice parcels are tracked in a Lagrangian framework to investigate how sea ice characteristics and their SEB co-evolve and respond to atmospheric conditions. Beginning on 1 October 2002, sea ice parcels are identified in 25-km grid cells where sea ice concentrations are > 15% and given a unique identification number. Adapting the Lagrangian approach^41^, the location of each sea-ice parcel is tracked daily using the weekly Simulated 12-month Ice Parcel Tracks from Gridded Sea Ice Motion Version 1^42^ on the 25-km Equal-Area Scalable Earth (EASE) Grid. These weekly ice motion data are linearly interpolated to daily vectors. Weekly ice motion data are used to reduce uncertainties associated with assimilating daily buoy motion in the product (personal communication with W. Meier, 2018). If sea ice concentrations fall below 15%, a parcel’s tracking is ceased. If more than 15% sea ice concentration materializes in open water grid cells, a new sea ice parcel is identified and tracked. It was found that the sea ice motion product can produce accurate tracking of parcels over time with little cumulative errors due to largely unbiased motion evaluations^43^. When comparing drift tracks to the drift of the Surface Heat Budget of the Arctic Ocean (SHEBA) ice camp, an error of 27 km over 293 days was found^41^. One study found that the standard deviation of the sampling error in ice motion to be 5 km to 12 km per day by comparing ERS-1 synthetic aperture radar (SAR) and drifting buoy motion to Lagrangian parcel tracks^44^. A separate simulation is run for each year, running from the beginning of October to the end of September of the following year (i.e., October 2002 - September 2003). Each year, new sea ice parcels are identified at the beginning of October. Sea ice parcels that did not melt out by the end of September are “linked” with the sea ice parcels identified the following October and are flagged as multiyear ice (see “Outputs from Database” section below for more details). At the time of writing, the database includes data through September 2020.

**Fig. 1:**
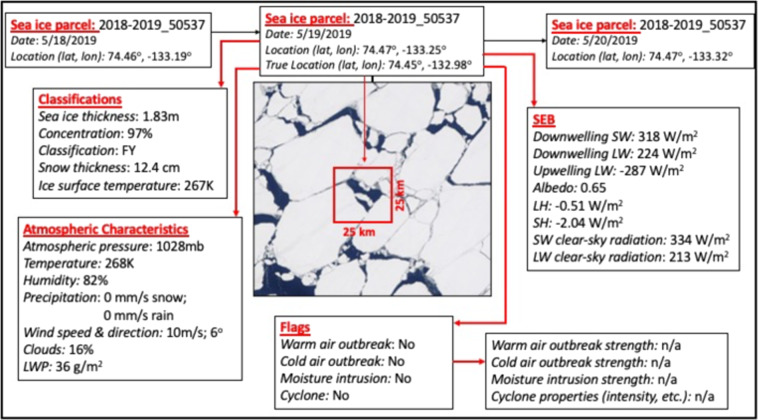
A schematic and details of the information associated with the Lagrangian tracked sea ice parcel #2018–2019_50537. Each sea ice parcel, on each day of the year, contains sea ice characteristics, atmospheric characteristics, SEB, and episodic weather cyclone event flags at a specific date and location. In the schematic, MY is multi-year sea ice, FY is first-year sea ice, LWP is liquid water path, SW is shortwave radiation, LW is longwave radiation, and LH is latent heat flux and SH is sensible heat flux.

At each time-step, daily averaged variables of interest are incorporated as individual data layers for each sea ice parcel including those characterizing sea ice conditions, the SEB terms between the parcel and atmosphere, and atmospheric conditions (see Table 1 and Fig. 1). All variables are re-projected to the EASE projection to match the coordinate reference system projection of the sea ice trajectories^45,46^. Along with these data layers, flags are given to denote the presence/absence of episodic weather events. Specifically, if a cyclone is present at the location of the parcel, the parcel is flagged with unique system identifications along with distance from the center of the system, the cyclone area, and several metrics of cyclone intensity. By incorporating synoptic event information, users can pinpoint perturbations that may result in propagating effects on the SEB and sea ice mass balance. Currently only closed system cyclones are flagged, however future work plans to include other types of episodic weather events.

**Table 1:** The following data are used for assembling the ice parcel database.

Variable	Data Source	Resolution
**Lagrangian Sea Ice Parcel Tracking**		
Sea Ice Drift	SSM/I PMW, Buoys^94^	Weekly, 25 km
Sea Ice Trajectories	Simulated 12-month Ice Parcel Tracks from Gridded Sea Ice Motion, Version 1^42^	Weekly, 25 km
**Sea ice conditions**		
Sea Ice Concentration (%)	SSM/I^48,49^	Daily, 25 km
Sea Ice Thickness (m)	PIOMAS^54^	Daily, 22 km
Snow Depth (cm) and Density (kg/m^3^)	SnowModel-LG^39^	Daily, 25 km
**Surface Energy Budget**		
Downwelling Shortwave (SW) Radiation (W/m^2^)	CERES^68,69,95^	Daily, 20 km
Upwelling and Downwelling SW and Longwave (LW) Clear-sky & All-sky Radiation (W/m^2^)	CERES^68,69,95^	Daily, 20 km
Albedo	CERES^68,69,95^	Daily, 20 km
Latent (LH)/Sensible (SH) Heat Flux (W/m^2^)	Derived from AIRS^72,73^	Daily, 25 km
**Atmospheric Conditions and weather event classification and tracking**		
Clouds fraction (%) and type (low, mid-low, mid-high, high), precipitable water (cm), liquid and ice water path (g/m^2^)	CERES-MODIS/ CALIPSO-CloudSat	Daily, 20 km
Atmospheric pressure (Pa), temperature (K), specific humidity (kg/kg), total precipitation (kg/m^2^), snowfall (kg/m^2^), total column water vapor (kg/m^2^), wind speed (m/s) & direction (°)	MERRA-2^57^	Hourly/3-hourly, daily, 1/2° × 5/8°
Atmospheric pressure (Pa), temperature (K), specific humidity (kg/kg), total precipitation (m), snowfall (m), total column water vapor (kg/m^2^), wind speed (m/s) & direction (°)	ERA5^58^	Hourly, daily, 1/2° × 1/2°
Atmospheric pressure (hPa), geopotential height (m), temperature (K), relative (%) & specific humidity (g/kg), skin temperature (K), surface air temperature (K), total precipitable water (kg/m^2^)	AIRS^60,61^	Daily, 25 km
Cyclone identification (#), distance from center of cyclone (km), cyclone area (km^2^), maximum Laplacian, maximum wind speed (m/s), minimum surface pressure (hPa)	The Melbourne University cyclone tracking scheme^62,65,66^	6-hourly, 1° × 1°

**Fig. 2:**
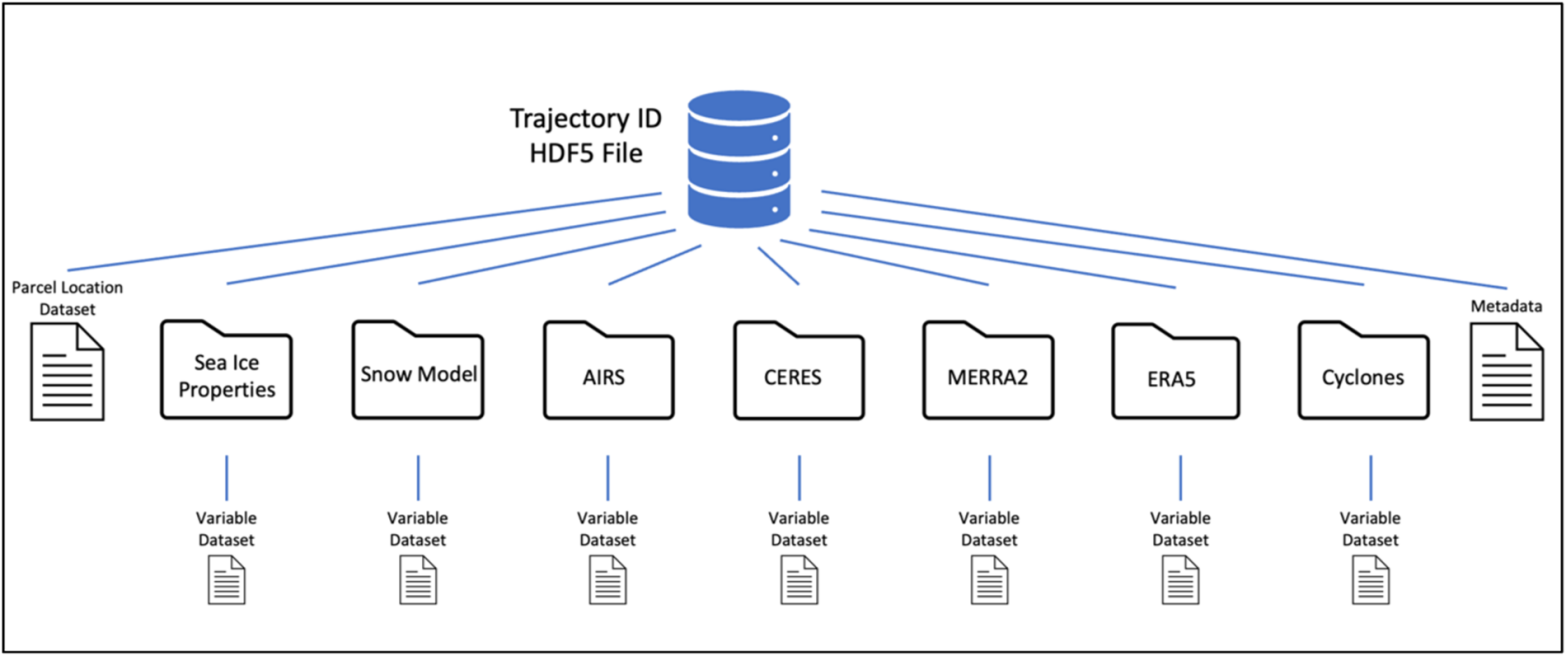
File structure for individual trajectories in the database.

**Fig. 3:**
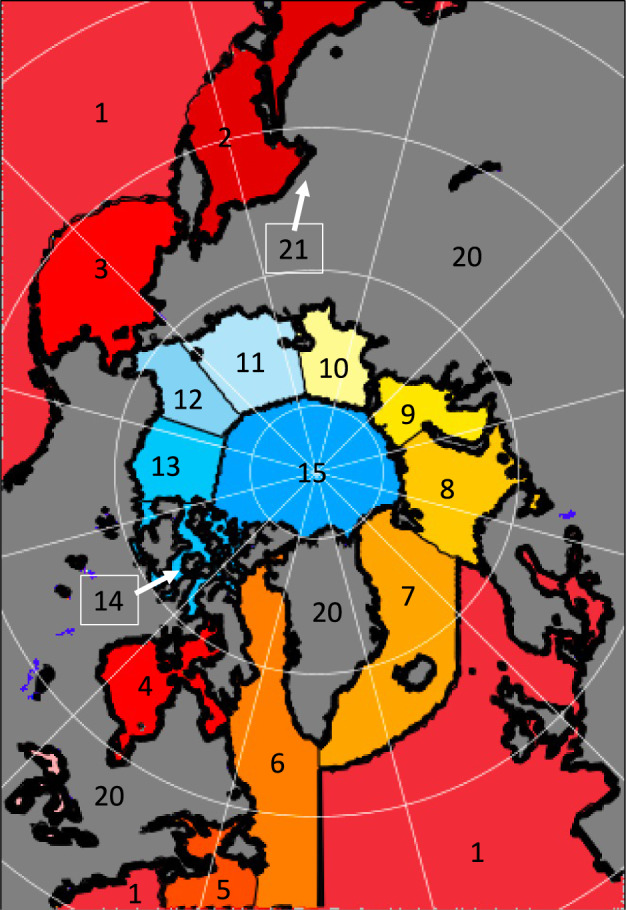
Database domain uses the 25-km polar stereographic grid EASE from NSIDC. Each number corresponds to a different region. These are, 1: Open Ocean, 2: Sea of Okhotsk, 3: Bering Sea, 4: Hudson Bay, 5: North Atlantic, 6: Baffin Bay/Labrador Sea, 7: E. Greenland Sea, 8: Barents Sea, 9: Kara Sea, 10: Laptev Sea, 11: E. Siberian Sea, 12: Chukchi Sea, 13: Beaufort Sea, 14: Canadian Archipelago, 15: Central Arctic, 20: Land, and 21: Coasts. These numbers are included in the metadata for each parcel.

The database is stored in HDF5 file format where an individual file exists for each unique sea ice parcel (Fig. 2). The top level contains the geographic location and date of the sea ice parcel at daily time steps. The ice parcel group level includes additional start and end regions (see Fig. 3 for a regional map) according to the updated region mask provided by the National Snow and Ice Data Center (NSIDC)^47^. A group exists for each data source that has been combined with the sea ice parcel (Atmospheric Infrared Sounder (AIRS), Pan-Arctic Ice Ocean Modeling and Assimilation System (PIOMAS), Modern-Era Retrospective Reanalysis Version 2 (MERRA-2), Cyclones, etc.). Every group contains a dataset for each individual variable, with multiple columns denoting pressure levels when applicable. The files are grouped by year (“year” here is from October 1 to the following September 30) and for each year a metadata file exists summarizing key characteristics of each sea ice parcel which can be used to filter sea ice parcels that meet certain criteria. Because the tracking algorithm is restarted each year at the beginning of October, parcel ID numbers do not carry over from September into October. Instead, an additional metadata file is included linking all parcels that survived summer melt (e.g., did not melt out by the end of September) with the nearest parcel at the beginning of the following October so that it is possible to track parcels across multiple years.

### Sea Ice conditions

#### Sea Ice concentration

Two sea ice concentration data products are included in this database, the Sea Ice Concentration Climate Data Record (CDR)^48^ (https://nsidc.org/data/G02202/versions/4| National Snow and Ice Data Center) and the Sea Ice Index product housed at the NSIDC^49^ (https://nsidc.org/data/G02135/versions/3). Both datasets are derived from two sources: (1) the Near-Real-Time Daily Polar Gridded Sea Ice Concentrations (NRTSI) from the Special Sensor Microwave Imager/Sounder (SSMI/S) on board the Defense Meteorological Satellite Program (DMSP) satellites^50^ and (2) the DMSP Special Sensor Microwave/Imager (SSM/I, 1987–2007), and the Special Sensor Microwave Imager/Sounder (SSMI/S, 2007 to 2019). The Sea Ice Index uses the NASA Team Algorithm^51^ for sea ice concentration estimates, while the CDR is a rule-based combination of the NASA Team Algorithm and the NASA Bootstrap algorithm^52^. Sea ice concentrations derived from the NASA Team algorithm are used to determine when to start/stop the Lagrangian tracking method described above. Multiple sources of sea ice concentration are included as different algorithms perform better than others in certain conditions (i.e., low ice concentrations), although the trends in sea ice area and extent tend to agree^53^.

#### Sea Ice thickness

Continuous, daily sea ice thickness estimates from observations are lacking for 2002–2020. Therefore, daily sea ice thickness is obtained from the Pan-Arctic Ice-Ocean Modeling and Assimilation System (PIOMAS), a coupled ocean and sea ice model that focuses on the Arctic Ocean^54^ (http://psc.apl.uw.edu/research/projects/arctic-sea-ice-volume-anomaly/data/model_grid). PIOMAS is driven by National Centers for Environmental Prediction (NCEP)/National Center for Atmospheric Research (NCAR) reanalysis data and is formulated in a generalized orthogonal curvilinear coordinate system which is used for bilinear interpolation with every sea ice parcel location. This grid has a mean horizontal resolution of 22 km for the Arctic Ocean. Sea ice thickness values produced by PIOMAS compare favorably with Ice, Cloud, and land Elevation Satellite (ICESat) measurements, with a correlation of 0.83 and root mean squared error of 0.61 m for spring (February-March), and a correlation of 0.65 and root mean squared error of 0.76 m for autumn (October-November)^55^.

#### Snow depth and density

As with sea ice thickness, continuous, daily snow depth estimates from observations are not available for incorporation into this product. Daily, pan‐Arctic snow depth and density on a 25-km × 25‐km grid are obtained from the Lagrangian snow-evolution model (SnowModel-LG)^39^ (https://nsidc.org/data/NSIDC-0758/versions/1). The model is forced with NASA’s Modern Era Retrospective‐Analysis for Research and Applications‐Version 2 (MERRA‐2) and European Centre for Medium‐Range Weather Forecasts (ECMWF) ReAnalysis‐5th Generation (ERA5) atmospheric reanalysis products, providing two individual sets of snow properties. By performing full surface and internal energy balances and mass balances within a multilayer snowpack evolution system, SnowModel-LG accounts for rainfall, snowfall, sublimation from static‐surfaces and blowing‐snow, snow melt, snow density evolution, snow temperature profiles, energy and mass transfers within the snowpack, superimposed ice, and ice dynamics. The redistribution of snow particles due to wind is not included in SnowModel-LG as the sea ice parcel sizes (14 × 14 km in SnowModel-LG) are too large to simulate snow erosion and deposition. Other possibly important processes that are not incorporated in SnowModel-LG include snow blowing into leads and snow-ice formation (when seawater floods the snowpack and refreezes due to a heavy snow load that submerges the ice surface below sea level). SnowModel-LG outputs have shown reasonable agreement with ice mass balance (IMB) buoys and measurements from the Surface Heat Budget of the Arctic Ocean (SHEBA) experiment and the Norwegian young sea ICE (N-ICE2015) measurements^56^.

### Atmospheric conditions

Inclusion of both Modern Era Retrospective Analysis for Research and Applications (MERRA-2)^57^ (https://gmao.gsfc.nasa.gov/reanalysis/MERRA-2/data_access/) and ECMWF Reanalysis 5th Generation (ERA5)^58^ (https://www.ecmwf.int/en/forecasts/dataset/ecmwf-reanalysis-v5) variables provides more flexibility in research applications, such as relating the database to output from models forced by different reanalyses. For instance, SnowModel-LG produces two sets of snow characteristics, one forced with MERRA-2 and one forced with ERA5. Inclusion of atmospheric variables from each reanalysis model enables consistency for snow-atmosphere comparisons.

#### European centre for medium-range weather forecasts (ecmwf) reanaylsis 5^th^ Generation (ERA5)

ERA5 was produced using 4D-Var data assimilation in CY41R2 of ECMWF’s Integrated Forecast System (IFS), with 137 hybrid sigma/pressure (model) levels in the vertical, with the top level at 0.01 hPa. Values have a spatial resolution of 0.25° latitude by 0.25° longitude. Surface values used here include total precipitation, snowfall, skin temperature, surface pressure, 2-meter air temperature, total column water vapor, and 10-meter wind speed and direction. Specific humidity, air temperature, and wind speed and direction at four pressure levels (1000hPa, 925hPa, 850hPa, & 500hPa) are also available. Although atmospheric data from ERA5 correlates well with *in situ* measurements taken during the N-ICE2015 campaign, ERA5 was found to have a large positive bias in 2 m temperature in winter and spring^59^.

#### Modern era retrospective reanalysis - version 2 (MERRA-2)

MERRA-2 uses the Goddard Earth Observing System, Version 5.12.4 (GEOS-5) atmospheric model and Global Statistical Interpolation (GSI) analysis scheme and has an approximate spatial resolution of 0.5° latitude by 0.625° longitude. Surface values used here include total precipitation, snowfall, skin temperature, 2-meter air temperature, surface pressure, total column water vapor, 10-meter wind speed and direction, total precipitable water, and total precipitable snow. Specific humidity and air temperature at two pressure levels (850hPa & 500hPa) and wind speed and direction at three pressure levels (850hPa, 500hPa, & 250hPa) are also available. Although atmospheric variables from MERRA-2 correlate well with *in situ* measurements during the N-ICE2015 campaign, MERRA-2 was found to have large biases in the total column water vapor in spring and summer^59^.

#### Atmospheric infrared sounder (AIRS)

NASA’s AIRS onboard the Aqua satellite was launched in May 2002 and has been collecting twice daily, global data ever since. AIRS has 2378 infrared channels and a 13.5 km spatial resolution. The AIRS instrument was designed to produce highly accurate temperature and humidity profiles globally^60,61^ (https://airs.jpl.nasa.gov/data/get-data/standard-data/), which is important in the Arctic where data are sparse and clouds are prevalent. AIRS Version 6 temperatures and humidity products have been compared with a variety of *in-situ* data and have shown to have modest errors in skin temperature (+/−2.3 K), 2 m air temperature (+/−3.41 K) and specific humidity (+/−0.55 g/kg)^19,25^. Version 7, AIRS-only atmospheric variables are used and include single level values of skin temperature, surface air temperature, and total column precipitable water as well as air temperature, geopotential height, and relative and specific humidity at 6 pressure levels (1000hPa, 925hPa, 850hPa, 700hPa, 600hPa, & 500hPa). Some of these variables are also provided by MERRA-2 and ERA5, but AIRS provides an observational perspective to complement the model derived variables.

#### Cyclones

The Melbourne University cyclone tracking scheme^62^ is used for identifying closed cyclone systems due to its consistency in capturing cyclone events, its broad agreement in results with other cyclone tracking algorithms^63,64^, and the availability of methodology from Webster *et al*.^65^. To describe the tracker briefly, sea level pressure (SLP) fields from ERA5 reanalysis are regridded and smoothed to 1-degree resolution on the polar stereographic grid as described by Murray and Simmonds^66^. The Laplacian (LP) of the SLP fields is then calculated to determine the local maxima of LP relative to eight neighboring grid cells. Once these local maxima are identified, a set of criteria are imposed: (a) the second derivative of the SLP in the x- and y-directions must be positive, and (b) the mean LP in the immediate vicinity of the maxima must meet the “concavity criterion” where LP is equal to or greater than 0.2 hPa per degree latitude squared. At every 6-hourly time-step, the cyclone centers are determined through an iterative approach that finds the minimum first derivatives (in x and y) within the local area of a center candidate, identifying both open and closed systems. The cyclone area is determined by fitting an ellipse to the near-zero LP values in eight opposing directions from the cyclone center. For the purpose of the ice parcel database, only closed systems are included and given unique identifiers representing individual systems. All points within the cyclone area are flagged as the same cyclone event, and if multiple cyclones overlap in a given area, those points are flagged with each cyclone ID. Along with the cyclone ID, the minimum SLP, maximum LP, maximum wind speed, distance to cyclone center, and cyclone area information are included in the ice parcel database as a group.

### Surface energy budget

#### Clouds and earth’s radiant energy system (CERES)

The CERES instrument is used by the Radiation Budget Science Project at NASA Langley to produce surface, atmosphere, and top-of-atmosphere radiative fluxes. These data products range from the instantaneous fluxes for each ~20 km CERES footprint to monthly, gridded radiative fluxes. This product incorporates CERES radiances, Moderate Resolution Imaging Spectroradiometer (MODIS) cloud properties, surface albedo retrievals, and meteorological information from the Global Modeling and Assimilation Office (GMAO) to produce 1-hourly resolved surface, atmosphere, and top-of-atmosphere radiative fluxes. Comparisons between the CERES longwave (LW) and shortwave (SW) surface fluxes and surface radiometer observations show uncertainties ~6% in the longwave and 23% in the shortwave at the hourly, regional time scale over global ocean and land^67^. Clear-sky/all-sky surface and top-of-atmosphere radiative fluxes are obtained from the CERES CERES-SYN1DEG product^68,69^ (https://ceres.larc.nasa.gov/data/#syn1deg-level-3).

#### AIRS-derived turbulent fluxes

The turbulent flux terms of sensible (SH) and latent (LH) heat are produced using AIRS Version 7 Level 3 data products of skin temperature, 925 and 1000 hPa air temperature, relative humidity, and geopotential height (https://airs.jpl.nasa.gov/data/get-data/standard-data/), MERRA-2 10 m wind speed and passive microwave sea ice concentration produced using the NASA Team algorithm^70^. Turbulent fluxes are estimated using the bulk aerodynamic method with the Monin-Obukhov Similarity Theory and an iterative calculation based on Launiainen & Vihma^71^ on the 25km^2^ polar stereographic grid. These fluxes are derived with a few modifications that were tailored specifically to capture the unique conditions of the boundary layer and roughness of the Arctic sea ice (see for more information^72,73^). The Arctic sea ice specific changes made to this algorithm have not been adapted or included in any other climate models or reanalysis products and are better suited to simulate turbulent fluxes from the Arctic Ocean. In fact, when compared with *in situ* data from the N-ICE2015 campaign, AIRS LH and SH fluxes had errors of 0.74 W/m^2^ and 5.32 W/m^2^, respectively^25^. Overall, these comparisons produce an error of ~20% in the AIRS-derived surface turbulent fluxes, but provide the most complete picture of Arctic surface turbulent fluxes over a 20-year period, in the absence of *in situ* data.

The database presented in this work is the first to incorporate the CERES surface radiative fluxes^68^ and AIRS surface turbulent flux data^19,25^. This enables the complete characterization of the SEB evolution of sea ice parcels across the Arctic domain.

## Data Record

The dataset is available at the National Snow and Ice Data Center (NSIDC) 10.5067/NJRT1HKVTFAQ^74^. This dataset contains yearly directories for 2003–2020, where each year begins on October 1 of the previous year (start year) and ends on September 30 of the year (end year). In each of these directories there is one file for each sea ice parcel in HDF5 format. Each file name is ‘TrajD_SYYY-EYYY_XXXXX.hf’, where SYYY is the start year and EYYY is the end year, and XXXXX is the unique trajectory number. Each trajectory file has information of the parcel’s latitude and longitude location at each day of its lifetime. Each file also contains sub folders for AIRS, CERES, ERA5, MERRA-2, PIOMAS, sea ice concentration, snow, and cyclone information for that particular parcel at each day of the parcel’s lifetime. Information about each variable in each subfolder is contained in the files, with long name and unit descriptors. There is also information on each parcel in the ICE-PARCEL subfolder which contains information on the duration, start and end regions and dates, and if it survives the summer melt or not.

### Validation of remote observations

Data from sea ice mass balance (IMB) buoys is obtained from the CRREL-Dartmouth Mass Balance Buoy Program^75^. These buoys were deployed in various regions throughout the Arctic Ocean and recorded sea ice thickness, snow depth, and air temperature and pressure along with GPS locations. IMB buoys provide 4-hourly data that are aggregated here to daily means. The ice mass balance buoy data are incorporated into the ice motion data^42^ of the ice parcel database and do not provide independent validation of drift location. However, the drift location of the buoys is useful for evaluating the derived Langragian sea ice parcel tracks. Additionally, comparing sea ice parcels with these buoys provides independent validation of key sea ice/snow/atmospheric variables.

## Technical Validation

### Trajectory evaluation

**Fig. 4:**
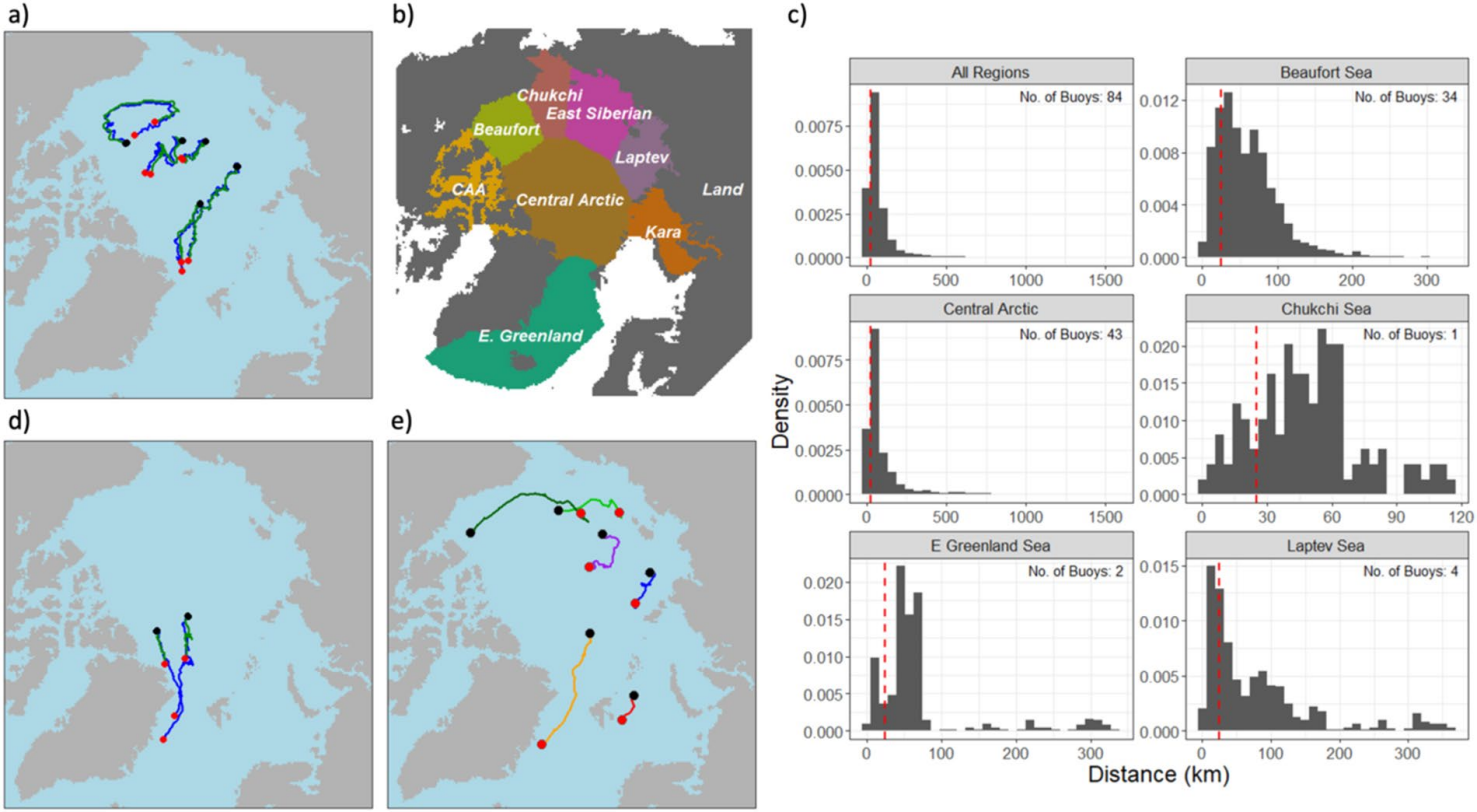
Comparison of ice mass balance buoy tracks and simulated sea ice parcel tracks. (**a**) Sample of buoy tracks (blue) and the closest ice parcel at time of deployment (green). The black/red dots represent beginning/ending locations. (**b**) Map of Arctic Ocean regions. (**c**) Histogram of the daily distance between buoys and ice parcel track by deployment region. Vertical dotted red lines indicate 25 km. (**d**) Buoy tracks (blue) and corresponding sea ice parcel trajectories (green) where the distance between tracks exceeds 500 km. Black/red dots indicate beginning/ending locations. (**e**) Selected example parcel drift tracks. Black/red dots indicate starting/ending locations.

The modeled sea ice parcel trajectories are assessed by comparing them to sea ice mass balance buoys that have been deployed throughout the Arctic Ocean^75^. Although IMB buoys are not an independent validation of the sea ice parcel drift locations, they are a useful tool for evaluating the results of the Lagrangian methodology. After removing buoys that do not match the time frame of our database and those that contain erroneous location data, we are left with 74 buoys that are used for comparison. Figure 4a shows the tracks of five buoys (blue) and the track of the closest ice parcel at the time of the buoy deployment (green). There are differences in daily positions, but overall, the derived trajectories closely match the buoy tracks. Sea ice parcels generally remain within 100 km of the corresponding buoy (Fig. 4c), with distances often much shorter (mean: 83 km, median: 54 km).

The buoy/parcel pairs originating in the Central Arctic and drifting south through Fram Strait show the largest discrepancy in ending locations, with the buoy traveling further south than the sea ice parcel (the date of the end point locations are the same for buoys and sea ice parcels as buoy drifts tend to last longer than that of the ice parcels). While many trajectories remain within 100 km of the associated buoy (Fig. 4c), those buoys deployed in the Central Arctic occasionally gain greater separation from the ice parcel, upwards of ~1,500 km. The buoy/parcel pairs that have distances greater than 500 km between them (Fig. 4d) are trajectories that begin in the Atlantic sector of the Central Arctic and drift south through the Fram Strait (only buoy locations with valid ice thickness measurements are shown as buoys can sometimes float in open water after the ice pack has melted). The buoys drift faster and further south along Greenland’s east coast than the simulated ice parcels, indicating that the ice motion vectors in Fram Strait do not capture the true ice velocities^76^.

### Mass balance and atmosphere comparison

**Fig. 5:**
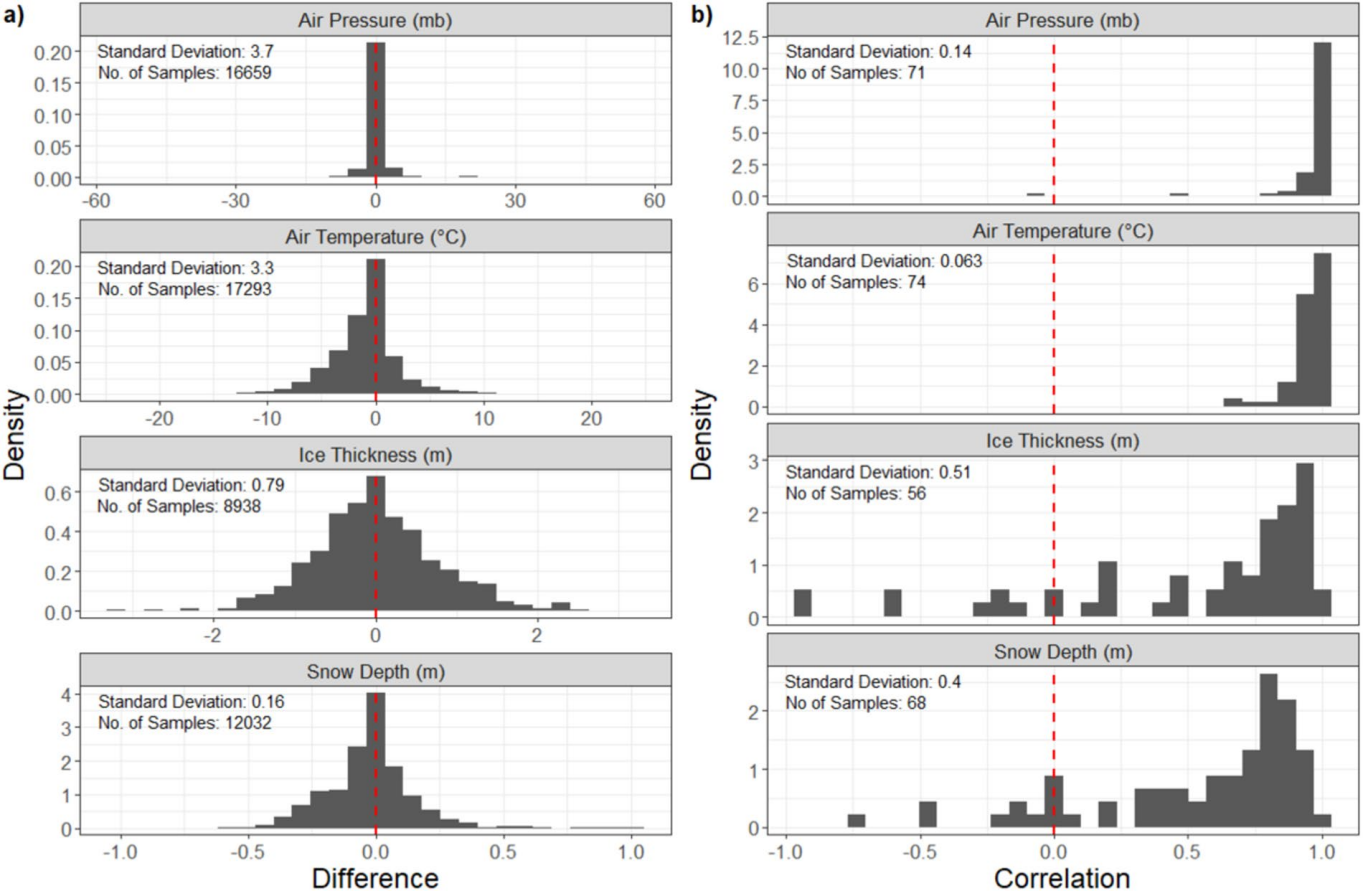
Comparison between buoys and tracked ice parcels of ice thickness, snow depth (from SnowModel-LG forced with MERRA-2), and air temperature and pressure (parcel air temperature and pressure are from MERRA-2). (**a**) Difference between buoy and ice parcel (buoy - ice parcel value), and (**b**) correlation between buoys and ice parcels (vertical dotted red line at 0 for both difference and correlation).

Comparisons of the properties observed by sea ice mass balance buoys and those derived from the sea ice parcel Lagrangian framework are shown in Fig. 5. Sea ice thickness and snow depth show the greatest variability compared to buoys (Fig. 5a). This can in part be explained by the spatial variability of these variables within each 25 km by 25 km grid cell. Because PIOMAS and SnowModel-LG provide averaged values for each grid cell, discrepancies between these values and *in situ* point sources (buoys) are expected. Using NASA’s Operation IceBridge^77^ as a reference, the standard deviation of sea ice thickness within a 25 km by 25 km grid cell on a given day ranges from 0.01–5.67 m, with a mean of 1.44 m and median of 1.43 m (data obtained from NSIDC’s IceBridge L4 Sea Ice Freeboard, Snow Depth, and Thickness, Version 1^78^). Smaller differences in air temperature and pressure are expected as these values have less spatial variability.

Comparing the sea ice parcel values with buoy data (Fig. 5b) provides an evaluation of the data set utility for addressing science related to the sea ice parcel evolution. All values show good correlation overall with a greater spread for sea ice thickness and snow depth. The mean correlation coefficients are 0.53 for sea ice thickness, 0.56 for snow depth, 0.95 for air temperature, and 0.96 for air pressure. Although there are some negative correlations for sea ice thickness and snow depth for individual parcels, collectively the sea ice parcels largely capture the evolution of the ice pack and are therefore a good source of information on assessing the evolution of the sea ice and snowpack along their drift trajectories annually.

**Fig. 6:**
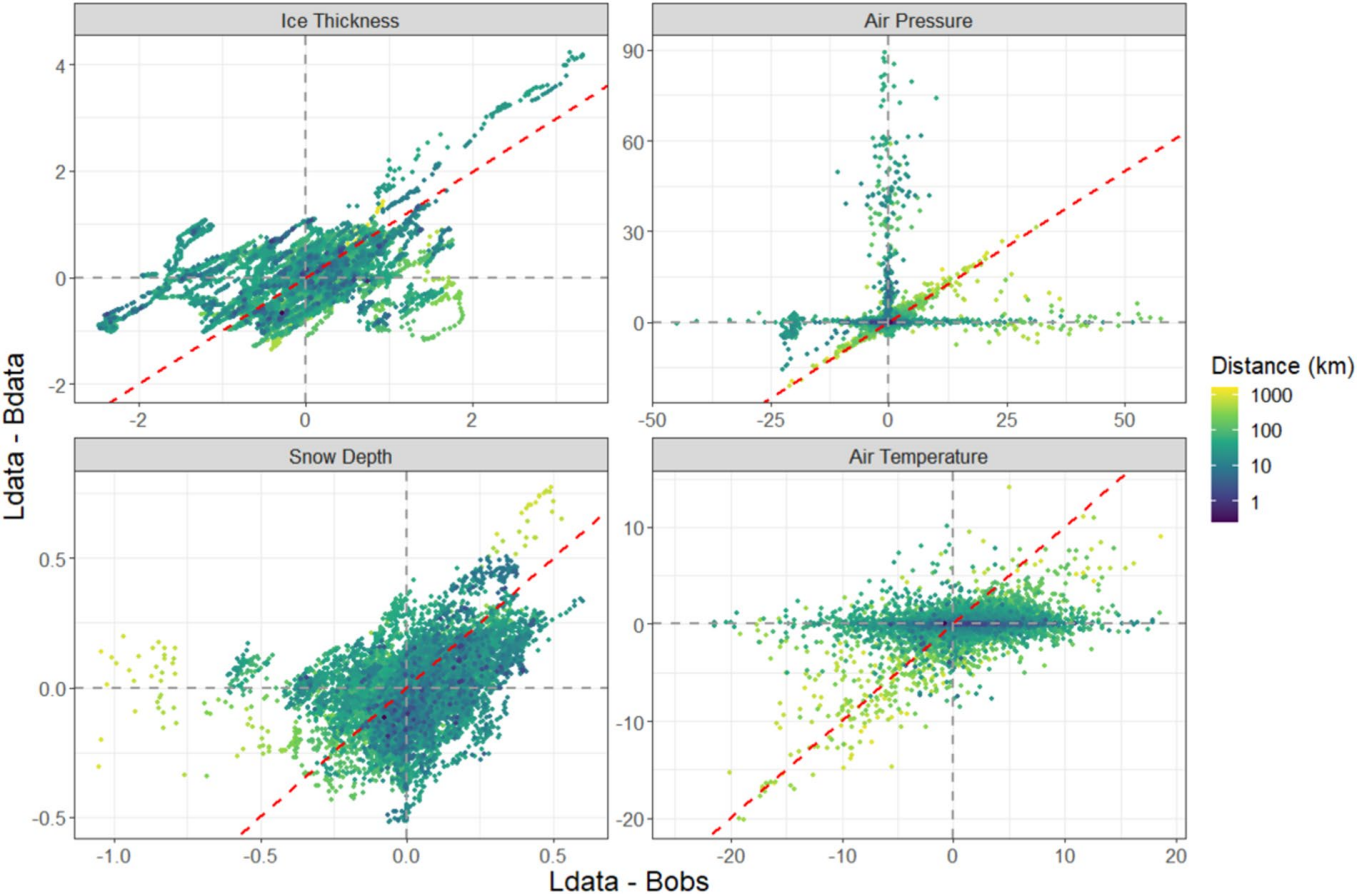
Influence of location errors on parameter errors. Color bar shows the distance between the Lagrangian track and the buoy (on logarithmic scale for clarity). Gray dotted lines mark zero for each axis, red dotted line shows the 1-to-1 line.

To evaluate how much of the discrepancies in the variables between the buoys and the dataset are due to errors in the Lagrangian tracking differences, due to deviating trajectories, are compared to differences arising from modeling/retrievals/sampling scale. This is done using the same methodology used for the Lagrangian tracked ice parcel database where common parameters from the input datasets are interpolated to the locations of the IMB buoys and averaged over the parcel area. This results in parameters produced in our Lagrangian tracks database (Ldata), parameters produced with the same methodology but with the buoy locations (Bdata), and the *in-situ* observations from the buoys themselves (Bobs). Figure 6 shows Ldata-Bdata vs Ldata-Bobs with points colored by distance between the Lagrangian track and the buoy. For air pressure, when ice parcels are large distances away from the buoy the primary driver of the difference is the distance between Lagrangian tracks and true locations as indicated by the points on the 1-to-1 line. Otherwise, when the points are not separated by large distances, the main source of error is due to modeling/sampling. For sea ice thickness and snow depth, inaccuracy of parcel location does contribute to parameter discrepancies, but this is true even at small distances as indicated by the roughly linear relationship regardless of distance. This suggests that the spatial variability of these variables is so large that some error will be present due to discrepancies in spatial sampling between a grid cell average and point measurement. Most of the difference in air temperature is due to modeling/sampling errors as indicated by the wide horizontal spread, little vertical spread, and well mixed distances. Therefore, we can conclude that location errors between the buoy and Lagrangian tracks are not the primary source of errors in the parameters.

The database trajectories and sea ice and atmospheric characteristics have been compared to data collected from sea ice mass balance buoys from the CRREL-Dartmouth Mass Balance Buoy Program^75^ by identifying the closest ice parcel to the deployment location. Results show ice parcels generally remain within 100 km of the corresponding buoy (Fig. 4c). The largest discrepancies are found near the Fram Strait where observed sea ice velocities tend to be much higher. Compared to the IMB data, the mean error of the ice parcel ice thickness and snow depth are typically greater than that of air temperature and pressure; this may be attributed to high spatial variability of the former two quantities when compared to a point measurement from a buoy. The overall high correlation coefficients between buoys and sea ice parcels show that changes in these quantities over time are in good agreement, suggesting the ice parcel database is useful for assessing sea ice evolution.

### Climatological studies

**Fig. 7:**
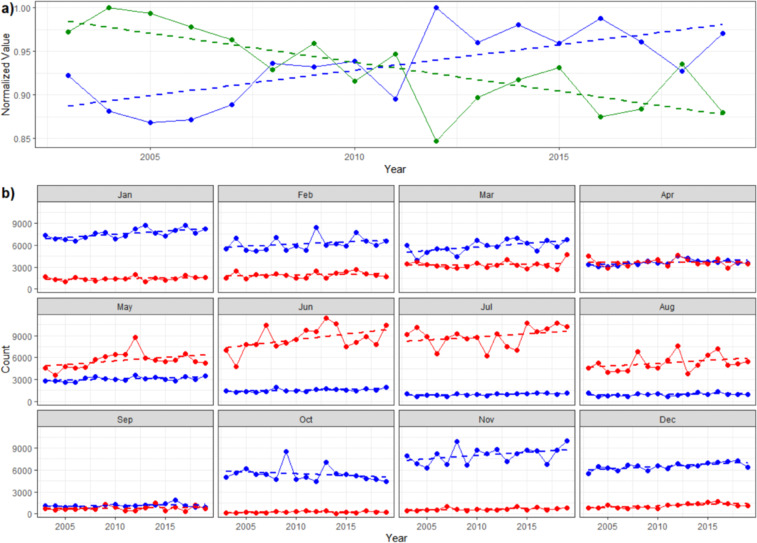
Yearly summaries of sea ice parcels. (**a**) Yearly total number of sea ice parcels (blue) and average duration of sea ice parcels (green). Both values are normalized for ease of comparison. Dotted lines are the linear fit. (**b**) Yearly number of sea ice parcels that melt (red) and freeze (blue) by month.

In addition to tracking the location and atmospheric - sea ice interactions for individual parcels, this database can be used to assess characteristics of sea ice parcels collectively. With declining sea ice cover in recent years, a reasonable expectation would be a decreasing number of sea ice parcels as well. However, the total number of sea ice parcels per year is increasing at a rate of 365.5 parcels or 228,437 km^2^ per year (Fig. 7a, blue). At the same time, the average duration (in days) of individual sea ice parcels is decreasing at a rate of -1.2 days per year (Fig. 7a, green). This demonstrates that the Arctic sea ice cover is transitioning to a more seasonal state with increased freezing and melting events, accounting for both the shorter duration of ice parcels and the increase in the total number of unique ice parcels. This result is consistent with the observed transition towards a seasonal sea ice dominated Arctic^2^.

To examine ice parcel freezing/melting events further, Fig. 7b shows the total count of parcel generation (freezing, blue) and extinguishing (melting, red) events by month and year. Except for freezing in October and melting in April, all trends are positive which can in part explain the simultaneous increase in sea ice parcels and decrease in sea ice parcel duration. Melting trends of 96.5 parcels (~60,312 km^2^) per year and 153 parcels (~95,625 km^2^) per year in May and June, respectively, are indicative of an earlier open water season in recent years^83^. Similarly, a negative trend in freezing sea ice parcels in October along with increasing trends in freezing in November (−50.4 (−31,500 km^2^) and 92.8 (58,000 km^2^) parcels per year, respectively) are representative of a later end to the open water season^79^.

**Fig. 8:**
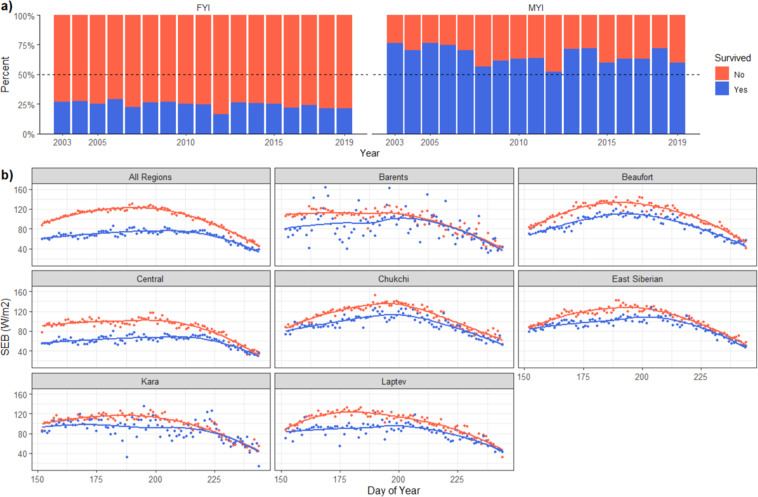
Survivability of sea ice parcels. (**a**) Percentage of first year (FYI) and multiyear (MYI) sea ice parcels that melt/survive (red/blue) the summer melt season. (**b**) Daily averaged net SEB for June-August, grouped by region where ice parcels end. Sea ice parcels that melted out are in red, sea ice parcels that survived the melt season are in blue. Lines are the locally estimated scatterplot smoothing (LOESS) curve fit.

With the shift from a predominantly multiyear ice (MYI) pack to a predominantly first year ice (FYI) pack in the ‘New Arctic’, the survivability of each of these ice classifications is of keen interest and can be observed as in Fig. 8a. The majority of FYI melts out every year while the majority of MYI survives the summer melt season. The interannual variability suggests this database can be used for case studies of particular sea ice years, such as the record low September 2012 extent, where there was a decrease in FYI and MYI that survived the summer melt.

The inclusion of CERES and AIRS data with these sea ice parcel trajectories provides opportunities to examine connections between the SEB and the fate of sea ice. As mentioned earlier, studies have shown that the SEB in June, July, and August can be a good predictor of September sea ice extent^29,80^. The SEB is calculated primarily with NASA remotely sensed observations of the radiative component from CERES and the turbulent flux component derived from AIRS:

1 $${\rm{Fr}}+{\rm{FL}}+{\rm{FE}}+{\rm{FS}}+{\rm{Fe}}={\rm{SEB}}$$

Where, Fr is the net absorbed SW flux, FL is the downwelling LW flux, FE is the upwelling LW flux, FS is the sensible heat flux and Fe is the latent heat flux. The conductive flux from the ocean through the sea ice is omitted here due to the lack of observational data. Figure 8b shows the daily averaged SEB for all sea ice parcels for these summer months, split by whether the sea ice parcel melted out (red) or survived the melt season (blue). In each region, the SEB was greater for sea ice parcels that melted out on average than those that survived. The greatest differences in SEB occurred between days 175 and 200 (late June through mid-July) which coincides with peak insolation.

### Case studies

**Fig. 9:**
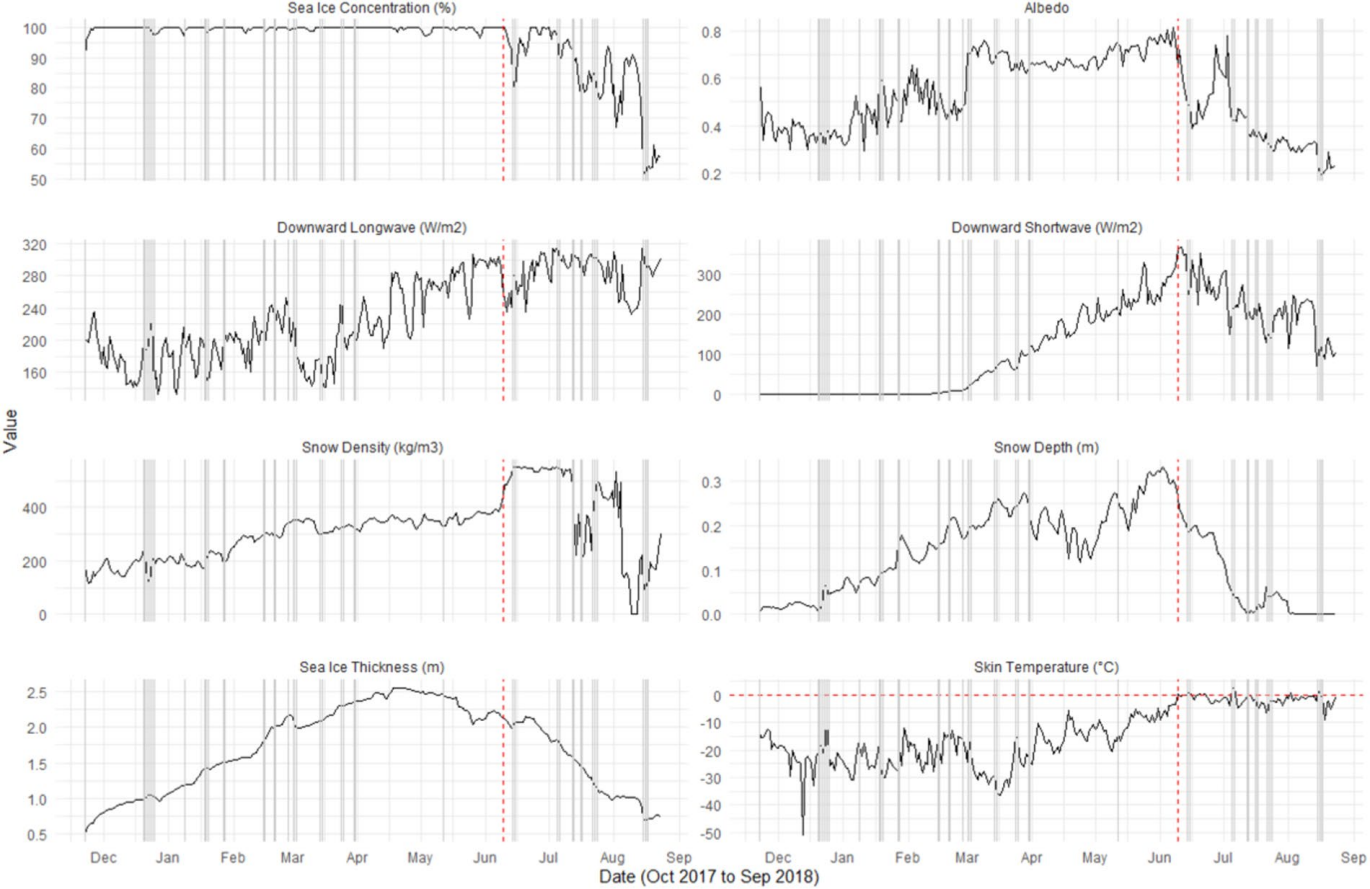
Variable time-series for the light green trajectory seen in Fig. 3e. Horizontal dotted red line indicates 0 °C. Vertical grey lines indicate cyclone flags. Vertical dotted red lines indicate the date skin temperature first rises above the freezing point. Sea ice concentration is from the sea ice concentration CDR, sea ice thickness is from PIOMAS, snow depth and density are from SnowModel-LG forced with MERRA-2, downward longwave and shortwave radiation and albedo are from CERES, and skin temperature is from AIRS.

Time-series of select variables relating to the SEB are shown in Fig. 9 for the light green trajectory seen in Fig. 4e that is advected from the northern Chukchi Sea into the East Siberian Sea. At the beginning of the second week in June (vertical dotted red line), after about a two-week period of consistently high downwelling longwave radiation, the skin temperature rises above the melting point and corresponds with a decrease in snow depth and albedo, and an increase in snow density. This could be an indicator of a melt onset event and the beginning of the melt season. Similar quick comparisons like this can easily be performed using this database of sea ice parcels and corresponding atmospheric conditions from October 2002 to September 2018. However, given the uncertainties of the input datasets, caution should be used in small scale (e.g., daily changes) analyses. This dataset is better used for larger temporal experiments, for example the cumulative effects of atmosphere-sea ice interactions over weeks/months/seasons.

**Fig. 10:**
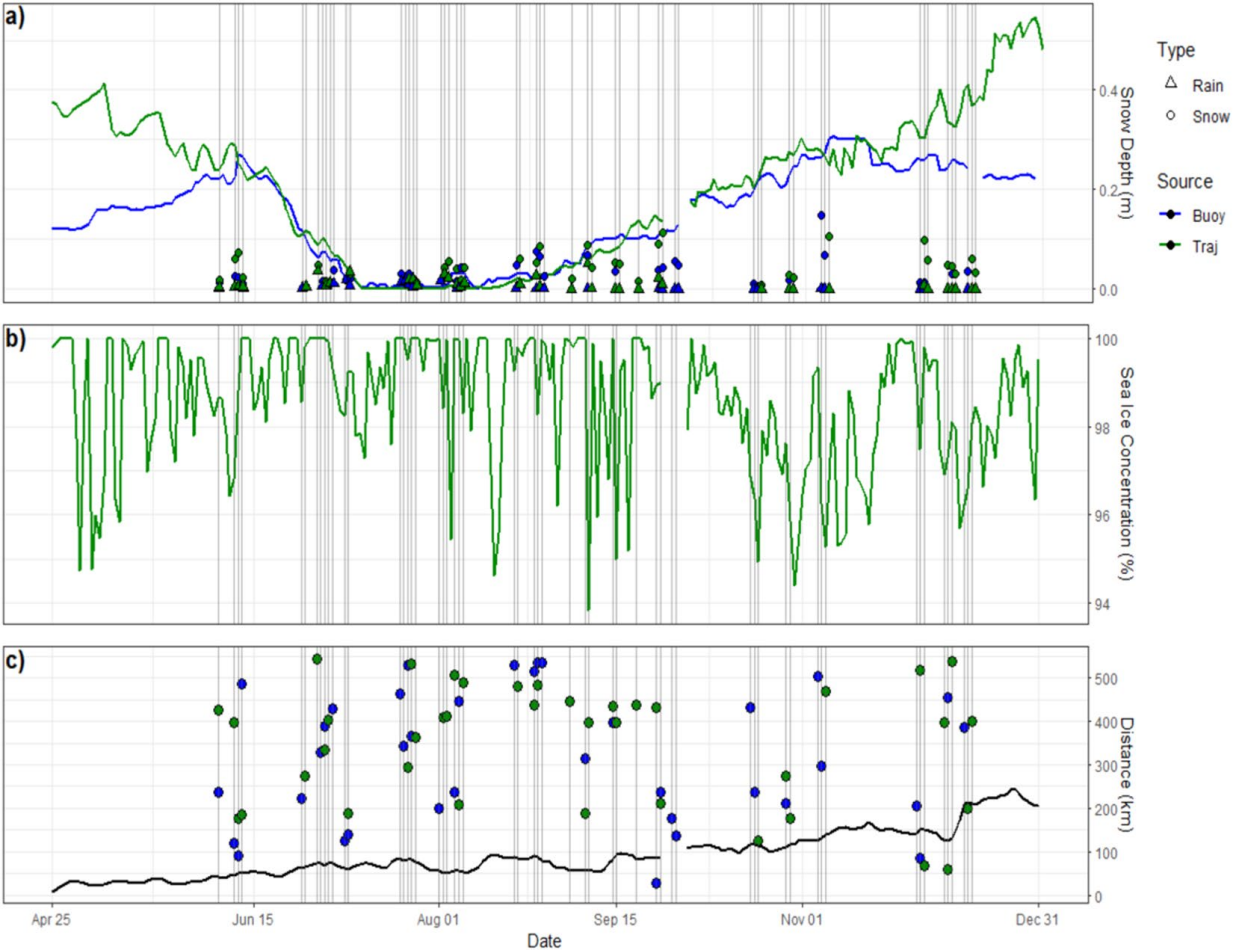
Comparison of buoy ID 2004D with the nearest sea ice parcel ID 2003–2004_19893 (orange trajectory in Fig. 3e). Blue/green represents values from the buoy/sea ice parcel database. Vertical lines indicate the presence of cyclones. (**a**) Snow depth measurements (sea ice parcel values from SnowModel-LG with MERRA2 forcing). Circles show daily total snowfall, triangles show daily total rainfall, both from the cyclone database. (**b**) Sea ice concentration (CDR). (**c**) Distance between the buoy/sea ice parcel and the center of the nearest cyclone. Black line indicates the distance between the buoy and sea ice parcel. The data gap at the end of September is the restart of the tracking algorithm.

Cyclone flags can be a useful tool for analysis of snow depth changes, precipitation events, and changes to sea ice concentration induced by sea ice thermodynamics. Figure 10 shows a sample time series from the CRREL-Dartmouth Mass Balance Buoy Program^75^ (buoy ID 2004D) and the nearest sea ice parcel at time of deployment (sea ice parcel ID 2003–2004_19893) where vertical lines indicate the presence of cyclones. As buoys/sea ice parcels can be influenced by multiple cyclones on the same day, only the nearest cyclone (distance from buoy/ice parcel to cyclone center) is shown. Because the buoy and sea ice parcel locations differ slightly they often experience different cyclone events (in Fig. 10a, blue/green circles & triangles show characteristics of the nearest cyclone to the buoy/ice parcel). Cyclones with large daily snowfall in spring after early June coincide with a slowdown of the generally decreasing snow thickness, while cyclones precipitating snowfall in the autumn are followed by a sharp increase in snow depth (Fig. 10a). Sea ice concentration tends to fluctuate regardless of whether a cyclone is in the vicinity, but rarely stays unchanged when a cyclone is present (Fig. 10b). Further analysis can examine relationships between changes to sea ice snow depth/density and sea ice concentration and the distance and direction of cyclones from sea ice parcels, and whether these relationships depend on the time of year.

General characteristics of collections of sea ice parcels can be determined with this database in addition to analyses of individual sea ice parcel time series. In recent years there has been an increase in the number of sea ice parcels that are formed and a decrease in the average duration of sea ice parcels suggesting more melt and freeze events (i.e., extinguishing and generation of sea ice parcels, respectively) and a transition to a seasonal sea ice cover. The survivability of sea ice parcels is linked to the June, July, and August summed SEB, where parcels that experience a larger flux of energy at the surface are less likely to survive the summer melt season. This can be expanded upon in future work by exploring the impact of individual components contributing to the SEB and the influence of SEB on autumn ice growth.

### Future additions

A ‘New Arctic’ sea ice parcel database has been presented which combines satellite observations and reanalysis data with daily sea ice parcel drift tracks produced in a Lagrangian framework. This novel dataset contains daily sea ice parcel locations, sea ice and snow conditions, and atmospheric states and fluxes from 2002–2019. Building on previous work^81^, this dataset includes drivers of surface energy fluxes from which the SEB can be calculated. Additionally, flags have been included to identify when sea ice parcels are potentially influenced by synoptic events such as cyclones. The dataset records distance from the center of the system as well as cyclone intensity. This dataset allows users to track the movement and evolution of sea ice parcels and the associated atmospheric state as they advect throughout the Arctic Ocean.

Current work is underway to include additional data layers to the Lagrangian ice parcel database for enabling studies of atmosphere-ice interactions; these data layers include: a daily surface melt and freeze product, indicators for extreme moisture and warm/cold air intrusions, and flags for polar low systems. Additional data sets may be added in the future where applicable, and the database will be updated yearly with current data.

The timing of melt onset and freeze up throughout the melt season is important for sea ice survivability throughout the year and in understanding the Arctic climate system^18,32,78,82,83^. Throughout the summer melt season, air temperatures tend to oscillate around the freezing point and there are multiple melt and refreezing events that occur at the surface^84^. A new daily melt onset data product (under development) can be incorporated to assess the amount of melt/freeze events that occur for individual sea ice parcels along with atmospheric and snow/sea ice conditions.

Future work will involve creating a database of extreme moisture and temperature intrusions and polar lows, and then applying these flags to the sea ice parcel Lagrangian database using a similar methodology to that used for cyclone events laid out in this paper. Extreme temperature and moisture intrusions will be identified using ERA5 and MERRA-2 atmospheric variables. Moisture intrusion events that enter the Arctic (crossing 70° N) are of interest as that energy can have significant impacts on the Arctic surface energy budget of the tracked sea ice parcels. To identify these events, a modified version of the methodology in Woods *et al*.^84,85^. and Woods & Caballero^86^ will be developed. This same criterion will also be used to identify warm and cold air outbreaks.

A database of polar lows will be created using ERA5 data and an adaptation of the cyclone tracking algorithm^62,65^. Polar lows are intense, mesoscale cyclones that are associated with fast propagation speeds; strong winds; high intensity precipitation as snowfall, hail, and/or rainfall; high waves; and freezing sea-spray^87,88^. Interactions between polar lows and the sea ice/ocean surface remain poorly understood and these events may have important implications for sea ice mass balance. Similar to the cyclone database, polar low flags will be added as a data layer to the sea ice parcels if they coincide with an event.

As satellite data of sea ice thickness have become more reliable, some of these datasets could also be incorporated into this data base. These include but are not limited to: the Soil Ocean and Ocean Salinity (SMOS)-CryoSat2^89^, CryoSat-2^90^, and the Ice, Cloud and land Elevation Satellite-2 (ICESat-2)^91^ thickness products to supplement the PIOMAS thickness data.

Once data from the Multidisciplinary drifting Observatory for the Study of Arctic Climate (MOSAiC) expedition has been quality checked and processed, MOSAiC datasets will present a unique opportunity to use *in situ* Lagrangian data to validate and interpret the snow depth and sea ice thickness results from the remotely-sensed ice parcel database presented here. MOSAiC was a year-long field campaign in the central Arctic where the R/V Polarstern was frozen into the pack ice^92^. The overarching objective of the MOSAiC expedition was to collect process-oriented, continuous field observations of the Arctic climate system year-round to advance understanding centered on Arctic system science in the ‘New Arctic’. The field experiments encompassed nested spatial scales up to 50 km and continuously drifted with the wind and ocean currents between Oct. 2019 – Oct. 2020.

The results shown here are just the ‘tip of the iceberg’ in the amount of new research and scientific results that this database will enable. This database has vast applications for the wider scientific community to utilize and to better understand sea ice-atmospheric interactions in the ‘New Arctic’ and to explore what atmospheric factors and their timing might hinder or aid in the survivability of sea ice throughout the year. It also enables process-oriented research when compared to previous Eulerian based investigations. This database could also be used to assess climate model simulations of Arctic variables and processes to evaluate and improve model physics. Currently, this dataset is expected to be hosted by the National Snow and Ice Data Center, where it will be available for public download.

## Data Availability

Code used for creating the Lagrangian sea ice parcel database were created using R coding software. This code can be accessed here: 10.5281/zenodo.7554521. Ice Mass Balance Buoy data which were used to assess the trajectories can be found here: https://imb-crrel-dartmouth.org/. Buoy Langrangian trajectories made with the database can be found here: 10.5281/zenodo.7554521^93^.
